# An Ongoing Teacher Professional Development Programme to Enhance Critical Health Literacy Pedagogies and Assessment

**DOI:** 10.1002/hpja.70016

**Published:** 2025-02-22

**Authors:** Louisa R. Peralta, Claire L. Marvell, James Barkell, Kellie Burns, Claire Otten

**Affiliations:** ^1^ Health and Physical Education, School of Education and Social Work, Faculty of Arts and Social Sciences The University of Sydney Sydney New South Wales Australia; ^2^ School of Medicine University of Tasmania Hobart Tasmania Australia

**Keywords:** adolescent, health education, health literacy, measurement, prevention, school

## Abstract

**Issue Addressed:**

Health literacy is an important asset for adolescents to develop through engagement in schooling and curriculum. The few studies that have focused on teachers, health literacy pedagogies and assessment, show that teachers find it difficult to enhance students' critical health literacy levels and to measure students' health literacy knowledge and capabilities using valid models. The aim of this study was to develop a longer‐term PD programme for secondary school teachers to enhance their ability to plan for critical health literacy learning and to co‐design with teachers a curricular model for assessing health literacy.

**Methods:**

Two face‐to‐face (F2F) PD sessions and two online PD sessions were scheduled with three participating specialist Health and Physical Education (HPE) teachers, seven HPE programmes were deductively analysed using Nutbeam's health literacy hierarchy and the Australian Curriculum: HPE outcomes and content.

**Results:**

Analysis showed that interactive learning activities were dominant (64%), compared with functional (4%) and critical learning activities (4%). The co‐designed curriculum model for measuring student health literacy was also developed for use in Australian schools. The resultant rubric is informed by Nutbeam's model, Broder et al.'s definition and Bloom's taxonomy.

**Conclusion:**

To our knowledge, this is the first ongoing teacher PD programme that has embedded co‐design processes for teachers and researchers to design a curricular health literacy assessment model for Australian and international HPE programmes.

**So What?:**

The valid measurement and assessment of child and adolescent health literacy has largely been ignored in previous research. This study is the first attempt to co‐design a curricular health literacy assessment for secondary schools that can be used by teachers and health professionals.

## 1 Introduction

The leading cause of death internationally are non‐communicable diseases (NCD) [1], with one‐third that can be prevented with early intervention [2]. Early intervention includes supporting the development of health literacy in schools and communities [3]. This was highlighted throughout the COVID‐19 pandemic, with the rapid rise of misinformation (easily propagated through technology and social media), which made health‐related choices challenging and disrupted NCD prevention, control efforts and exacerbated health inequity worldwide [1]. Developing health literacy can support individuals, especially young people, to make educated and autonomous health‐related decisions for themselves and others [4].

Health literacy has two interrelated components: individual health literacy and the health literacy environment [5]. Individual health literacy refers to one's ability to find, understand and communicate health information, including the capacity to make critical judgements about health claims [6]. Health literacy capabilities can be used to empower individuals to make informed health decisions, practice healthier behaviours and modify personal determinants of health [7]. The health literacy environment refers to the external supports around an individual that facilitate them to make educated and autonomous health‐related decisions [5]. For instance, school health education, health and physical teachers and the school environment supporting active play and respectful relationships in non‐curriculum time (i.e., at recess and lunchtime). Individual health literacy and the health literacy environment are both important and are the focus of this study.

An important life period to promote health literacy development is childhood and adolescence [8]. While many conceptualisations and definitions of health literacy focusing on children and adolescents exist [9, 10], there is a lack of consensus on an overarching conceptual model of health literacy for children and adolescents [11]. This has led to confusion and disparate efforts previously. Although there is a lack of consensus, what is known is that public health interventions (inclusive of health education programmes) are best when introduced early in life [1]. It has been suggested that promoting health literacy development during these formative years, has the potential to impact health across the lifespan [12]. Consequently, promoting health literacy development during this period is paramount.

The WHO has recognised health literacy as a critical determinant of health that must be an integral part of the skills and competencies developed over a lifetime, first and foremost through the school curriculum [13]. Schools have long been identified as appropriate settings for health promotion and health education [14, 15], since they can reach almost all school‐aged children regardless of determinants and background. Schools in the United States [16], Australia [17], Scotland [18] and Finland [19] have included health literacy in the curriculum. However, it is important to note that for United States and Finland, health literacy is an expected learning outcome of health education curriculum, or one of the primary goals of health education [20], whereas health literacy is an underlying proposition of the national curriculum for Health and Physical Education (HPE) in Australia [17]. This means that the programming and planning, teaching and assessment across international health education curricular may take different forms due to the explicit and implicit place of health literacy, but emphasises the central role that Australian HPE teachers have in promoting children and adolescent health literacy.

The conceptualisation of health literacy in the Australian Curriculum for HPE (AC:HPE) follows Nutbeam's [6, 21] hierarchical model consisting of three dimensions: functional, interactive and critical health literacy. This model characterises health literacy from an asset‐based perspective that is developed in phases through health literacy dimensions. These dimensions start with the basic literacy and numeracy skills required to find and understand basic health information (functional), progressing to the ability to interact, communicate and apply learned health information to change behaviour (interactive), and then the ability to evaluate and reflect upon information to make decisions about health at an individual and community level (critical) [6]. These three levels are embedded throughout the AC:HPE from Kindergarten (5–6 years old) through to Year 10 (15–16 years old). Therefore, the ability of schools, school principals, other school‐based leaders and HPE and generalist teachers to advance health literacy through curriculum, teaching, learning and assessment across the whole school environment is central to the implementation of health literacy education in schools and the promotion of children and adolescent health literacy in Australia [22, 23, 24]. However, studies focusing on HPE and generalist teachers' capabilities to support, teach and assess students' health literacy are few [25, 26]. Of the Australian studies that have focused on developing professional development (PD) programmes for Kindergarten (K) to Year 10 teachers focus on curriculum, programming, teaching and learning [24, 27, 28] and have acknowledged that most school‐based health literacy research has not focused on assessment or measurement of health literacy.

Recent research in Australia, across both primary (K to Year 6) and secondary schools (Years 7–10), has shown that HPE and generalist teachers need and want an ongoing PD programme to plan for and enhance students' health literacy levels [24, 27, 28], particularly, students' critical health literacy. Critical health literacy is an important facet of building children and adolescents' health literacy capabilities (as noted in Refs. [6, 21] hierarchal model and in the AC:HPE), as it encompasses actions that develop personal skills, build health public policy and supportive environments which are explicitly mentioned in the Ottawa Charter for Health Promotion [29]. In addition, Sykes and Wills' [30] assert that critical health literacy ‘encompasses both action and agency: to apply information critically, but crucially also to individual and political action on the social determinants of health in pursuit of social justice’ (p. 49). Given the sociocritical, strengths‐based nature and the rationale, aims outcomes and content of the AC:HPE, it is essential to explore the extent to which Australian teachers and school‐based health education programmes offer opportunities for children and adolescents to build upon their assets and capabilities to take individual and collective action in their own school and in their local and community settings [17]. Without measurement or assessment of health literacy using valid tools that reflect the contexts of learning (i.e., embedded in health education programming and implementation), it will be unclear whether schools and teachers of curriculum and pedagogy can influence critical health literacy development.

Drawing on Bernstein's [31] three message systems of education (curriculum, pedagogy and assessment), Hay and Macdonald [32] view assessment in health education as a message system. This perspective argues that assessment is a fundamental dimension of the selection, classification, transmission and control of programming and educational knowledge, including health literacy knowledge. Internationally, health education teachers have been challenged by changes in curriculum and to design appropriate teaching, learning and assessment strategies [33]. It is not surprising that Australian teachers responsible for teaching and assessing health literacy through AC:HPE programming and subsequent state‐based curriculum (e.g., NSW Personal Development, Health and Physical Education [PDHPE] syllabus [34]) also report the need for ongoing PD and guidance in this area [24, 27, 28].

Previous research has used health literacy questionnaires that have been designed for adults or have been derived from adult questionnaires. These traditional and summative evaluations of learning are restrictive as they focus on individual capabilities and on reporting high and low levels of health literacy at a given time‐point [35, 36, 37]. This has been supported in a systematic review of 21 studies [27], which found that health literacy questionnaires were mostly used to assess individual health literacy of children and adolescents, with those based on multiple choices questions being limited as they tend to focus on functional health literacy (basic skills in reading and writing) and participant characteristics of developmental change (of cognitive ability) [27, 38]. Health literacy questionnaires are not valid indicators for children and young adolescents (i.e., as they cannot accurately reflect their understanding) and tend not to embed the health education curriculum in which health literacy learning in schools is usually situated [9, 38]. As such, Peralta et al. [24, 28], Nash et al. [27], Otten et al. [26] and Summanen et al. [39] have emphasised that future school‐based health literacy studies need to develop a curricular model of health literacy that can be co‐developed, implemented and utilised by teachers.

Although there are varying degrees of engagement in participatory research [40], co‐design presents an opportunity for meaningful end‐user engagement throughout the health literacy research process [41]. Co‐design has been used globally with teachers who are responsible for teaching health education [42, 43], with one health literacy study utilising students to inform health education pedagogy [44]. Each of these studies show that the co‐design process empowers participants to actively engage in the design and development of intervention strategies, which may increase the likelihood of acceptability, effectiveness and sustainability of the resulting health education programme. Adopting a participatory approach allows for research to be carried out ‘with’ rather than ‘on’ participants. For example, teachers and external stakeholders (e.g., researchers) participate in design or problem‐solving processes, where all ideas and knowledge are equally appreciated throughout the process. As a result, curriculum programmes and assessment are more likely to be contextually relevant, feasible and sustainable. This highlights the need for PD programmes to embed co‐design opportunities for researchers and teachers to collaborate on pedagogies and assessment strategies that enhance students' health literacy learning through health education.

The aim of this study was to build upon previous research conducted in Australian secondary schools and with specialised HPE teachers (i.e., those that have the primary responsibility of teaching HPE) [24, 28] to: (1) develop a longer‐term PD programme for secondary school teachers to enhance their perceptions to plan for critical health literacy learning and (2) co‐design with teachers a curricular model for assessing health literacy. To our knowledge, this is the first ongoing teacher PD programme that has embedded co‐design processes for teachers and researchers to design and validate a curricular health literacy assessment model for HPE programmes.

## 2 Methods

### 2.1 Design

A single school case study was used.

### 2.2 Participants

The recruitment of participants occurred through convenience sampling [45], where school and colleague contacts of the research team were utilised. A total of *N* = 3 HPE specialist teachers from one school in Sydney, NSW, participated in the ongoing PD programme. The school is an independent, coeducational early learning and K‐12 within New South Wales, Australia. It is a metropolitan school of over 1000 students with an Index of Community Socio‐Educational Advantage (ICSEA) of over 1200 (i.e., the median ICSEA for all of Australia is 1000. Schools with a lower ICSEA [< 1000] are more disadvantaged. Schools with a higher ICSEA [> 1000] are more advantaged). The recruitment of teacher participants needed to include the HT HPE to ensure that programmatic changes would occur after the first PD session (during the extended school break) and before the second PD session, as those roles include leading staff through curriculum, programme review and writing roles.

### 2.3 Procedure

School recruitment began in June 2022. The project was advertised via existing school contacts and networks among the researchers. In June, one school expressed interest via email and provided the name and email address of the Head Teacher (HT of HPE; otherwise known as the lead curriculum teacher) of HPE. Communication between researchers and HT of HPE during June to October 2022, centred on coordinating one 40‐min PD session between October to December 2022, and one 30‐min PD session in March 2023. In the lead up to the first 40‐min PD session, the HT of HPE was asked to share a minimum of four of the school's HPE programmes (i.e., a unit of work), one programme for each year group (Years 7–10; students aged 12–16 years) that was developed by the HPE teachers to be implemented in 2023. It is important to note that Years 7 and 8 work towards one set of outcomes and Years 9 and 10 work towards another set of outcomes (i.e., these year groups span two set of outcomes).

Following the co‐design curriculum planning model posited by Kelly et al. [46], which has been tested in Australian schools and with Australian teachers, the ongoing PD programme and co‐design process was organised through two face‐to‐face sessions and ongoing support through implementation phases via email and Zoom. This was to optimise participation from all of the HPE teachers and researchers, using an action research approach [47] and to work through learning cycles until full consensus about the rubric were achieved. After the school and HPE teachers consented to participate in this study, and shared seven HPE programmes delivered in 2022, an initial 40‐min PD session was planned and delivered in December 2022. This session began with introducing the PD programme, aims and the co‐design process. Once this was clear and endorsed, personalised feedback was provided to the HPE teachers (*n* = 3) by two researchers (L.R.P. and C.L.M.), based on the health literacy content and pedagogies currently embedded in their HPE programmes that were delivered in 2022 (for more details of this approach see previous research conducted by [24, 28]). The session included co‐sharing of ideas on how to enhance the content and pedagogies in each of these programmes based on the analysis, with a particular focus on promoting critical health literacy in HPE programmes to be delivered in 2023. In addition, the teachers and researchers discussed what an assessment rubric for measuring health literacy knowledge and capabilities underpinning the HPE curriculum would look like with examples of current rubrics utilised at the school.

At the completion of the first PD session, the second 30‐min PD session was planned for March, 2023, to allow teachers time to make the changes that were suggested in the first PD session, and for researchers to design a health literacy rubric based on previous discussions and mapped against the health literacy content in the syllabus. The second PD session focused on reviewing changes made to the 2023 HPE programmes and working with the draft assessment rubric. In this session, further refinements were made to the assessment rubric to increase useability based on terminology and clarity, applicability to a range of tasks and ensuring an appropriate grading scale (i.e., the scale used at the school). After the second PD session, the HT of HPE and HPE teachers scheduled PD sessions via Zoom (*n* = 2, 15 min in length) during April to May, 2023, to discuss minor changes to be made to the health literacy promoting pedagogies and assessment rubric. The PD programme and co‐design process is outlined in Table 1.

**TABLE 1:** Ongoing PD programme sessions and co‐design processes and activities.

	Schedule	Processes and activities
Initial PD session (F2F)	December, 2022 (40 min)	PD programme outline, aims, co‐design process, HPE programme analysis results, co‐design pedagogies/activities based on results, HPE programme re‐design, introduction and initial discussion on HL assessment rubric
Co‐design session (F2F) #2	March, 2023 (30 min)	HPE programme co‐design discussion, sharing draft of co‐designed HL assessment rubric, practice using rubric on assessment tasks embedded in HPE programme co‐design
Ongoing PD sessions (online)	March–May, 2023	Consolidate HPE programmes and assessment rubric

### 2.4 Programme Analyses and Co‐Design Process

In line with Abel's [48] and Paakkari and Paakkari's [20] contention that the promotion of health literacy must be adjusted to the specific setting and educational aims, we aligned Nutbeam's three‐level framework with the outcomes, key inquiry questions and content, which has been published previously [24, 28]. These were then used to analyse the extent to which each level of heath literacy was promoted in the lesson activities for each of the seven HPE programmes. This data allowed for the creation of understandings of HPE teachers' abilities to plan and programme for health literacy, focusing on developing students' levels of critical health literacy.

We deductively analysed [49] each of the seven programmes (one for Years 7 and 8, two for Year 9 and three for Year 10) which together had a total of 223 learning activities. Each learning activity was categorised according to Nutbeam's [6, 21] health literacy hierarchy (i.e., functional, interactive or critical). The HPE programmes contained outcomes, key inquiry questions, content, learning activities and resources, which varied in length with an average of 15 pages (minimum of 7 pages and a maximum of 20).

Following initial conceptualisation discussions with all authors, two of the authors (L.R.P. and C.L.M.) analysed the learning activities individually, according to the hierarchy of functional, interactive and critical. Each HPE programme took the independent reviewers between 15 and 20 min to analyse. If the development of critical literacy was evident in the outcomes and learning activity, along with interactive health literacy, the activity was coded as critical literacy. Hence, the highest level evident was the code assigned to that activity (see Table 2). Once the authors had coded for all learning activities, they sent their coding independently to another author (J.B.), who was blinded to the analytic process. This co‐author performed an inter‐rater reliability check on the accuracy of coding by comparing her assessment with the initial coding results. Inter‐rater reliability checks led to an overall 88% agreement on coding allocation. Previous studies have identified 70% as the minimum acceptable level of agreement [50]. Discrepancies in coding were then analysed individually by the co‐author. The results of the second analysis were then discussed and refined in successive meetings before finalisation of the codes.

**TABLE 2:** HPE programmes and health literacy across Years 7–10 in the case study school.

School	Year 7 (*n* = 36)		Year 8 (*n* = 20)		Year 9a (*n* = 48)		Year 9b (*n* = 17)		Year 10a (*n* = 25)		Year 10b (*n* = 28)		Year 10c (*n* = 48)	
HL code frequencies of activities in programmes														
	%	(*n*)	%	(*n*)	%	(*n*)	%	(*n*)	%	(*n*)	%	(*n*)	%	(*n*)
Functional	19.4	(7)	17.4	(4)	22.9	(11)	53.0	(9)	64.0	(16)	25.0	(7)	12.5	(6)
Interactive	77.8	(28)	73.9	(14)	70.8	(34)	47.0	(8)	36.0	(9)	67.9	(19)	70.8	(34)
Critical	0.0	(0)	8.7	(2)	4.2	(2)	0.0	(0)	0.0	(0)	0.0	(0)	16.7	(8)
None	2.8	(1)	0.0	(0)	2.1	(1)	0.0	(0)	0.0	(0)	7.1	(2)	0.0	(0)

*Note:* Each activity in each unit plan was coded. Activities pertaining to assessment or those with insufficient information were not coded (e.g., ‘None’).

To aid and enhance the co‐design process, the research team conducted content analysis of the observation notes taken by the lead researcher to record the discussions that took place in each PD session. First, HPE teacher perceptions were identified within each session, which were presented back to participants towards the end of the session. These points were interpreted, critiqued, additions made and agreed upon by teachers themselves to promote authenticity of research as the HPE programmes were redesigned and curricular assessment model developed [51].

## 3 Results

The results are presented in two sections. The first section reports on the analysis of the seven HPE programmes that were presented in the first PD session, as well as the content analysis of the co‐design discussions that took place. As the HPE programmes were continually being developed as they were being taught in 2023 (as part of the ongoing PD programme), a secondary analysis of the HPE programmes was not deemed appropriate at the time. The second section presents the curricular model for assessing health literacy, as a result of the co‐design process.

### 3.1 Programme analysis

Findings indicate that the school aimed to implement health literacy through a selection of Years 7–10 HPE programmes and learning activities (to be taught during 2023). As displayed in Table 3, many learning activities were categorised as interactive (*n* = 63.5%), with a much smaller number of learning activities categorised as critical (*n* = 4.2%). The learning activities categorised as functional were 30.6%. For this school, one of the Year 10 programmes had the larger number of learning activities that had the potential to enhance students' critical health literacy levels (*n* = 16.7%), which were followed by a Year 8 (8.7%) and Year 9 programmes (2.1%). Four of the seven programmes had no learning activities that could enhance students' critical health literacy levels.

**TABLE 3:** Models informing the health literacy taxonomy.

Nutbeam	Functional HL		Interactive HL			Critical HL	
AC:HPE	Identify, recognise, describe, explore, explain		Communicate, plan, perform, refine, distinguish, implement, select and use, manipulate			Critique, investigate, examine, evaluate, assess, research, propose, create, adapt, transfer, design, reflection	
Revised bloom	Remember	Understand	Apply	Analyse		Evaluate	Create
Broder	Understood		Access	Use	Communicate	Appraise	

#### 3.1.1 Content Analysis of Observation Notes for the First PD Session

The three teachers involved in the first PD session appreciated the programme analysis of their HPE programmes and the simplicity of representing each activity as either functional, interactive and critical. However, the teachers also highlighted the need to represent activities in their programming in more detail, including teacher‐led questions and the scaffolding of student interactions and discussions. Teachers believed that this would have helped with determining whether critical health literacy would have been attained more often in discussion‐based activities that were littered throughout their HPE programmes. Adding this level of detail was an aspect that teachers wanted to spend time enhancing in their HPE programming for 2023.

The teachers also reported appreciating the time co‐designing with researchers to enhance several interactive health literacy learning activities in each HPE programme to become critical health literacy activities. As teachers know their students, how they learn and the contexts in which their students reside, the teachers and researchers worked through several different examples for each of the selected interactive activities to allow teachers a level of autonomy and flexibility with determining how critical health literacy learning would be best used in future HPE programming. This personalised approach was valued by the three HPE teachers and was highlighted as the most important component of the first PD session.

Finally, the teachers shared with researchers the assessment rubrics that they had designed previously and had been using to assess their students' physical literacy. These rubrics were discussed, with teachers wanting a similar approach for measuring students' health literacy for two main reasons: (1) for ease of use for teachers and (2) for ease of understanding for students as these rubrics had been unpacked and discussed with students previously. As such, the researchers used the HPE teachers' physical literacy rubrics as a basis for the health literacy rubric that would be drafted and shared in the second PD session.

### 3.2 Curricular model for assessing health literacy

Educational context taxonomies are useful as they classify learning in relation to level of task, knowledge or skill complexity [52]. The researchers drafted a curricular model for assessing health literacy using Nutbeam's [6, 21] hierarchal health literacy model (as it is this model that underpins health literacy knowledge and capabilities in the AC:HPE curriculum), Broder et al.'s [53] *Children and Youth Proposed Target‐Group‐Centred Definition*, and Bloom's (2001) *Revised Taxonomy of Educational Objectives* (shown in Table 3). The idea that Bloom's taxonomy and Nutbeam's taxonomy align with one another has been recognised previously [54], and recently, Broder's taxonomy and the World Health Organization's definition of health have been aligned with these existing models [55]. Recognising that health literacy development occurs in alignment with these taxonomies helps to understand the order in which capabilities are developed [55].

As health literacy knowledge and capabilities are demonstrative or performative, particularly, at the higher levels of interactive and critical health literacy, assessment of health literacy should reflect students' abilities to solve real‐world problems, analyse and synthesise information and apply their knowledge and skills. To do this reliably and objectively, a carefully designed scoring procedure with clear criteria is needed. Rubrics with criteria and performance level descriptions are deemed an effective vehicle for organising and communicating criteria, demonstrative and performance expectations and for use in scoring and/or providing feedback on student work [56]. There are several benefits of using rubrics: (1) increase consistency of judgement when assessing performance and authentic tasks. Rubrics are assumed to enhance the consistency of scoring across students, assessments, as well as between different raters, which is important in teaching when often there are more than one teacher teaching a HPE programme, across classes and Year groups [57, 58]; (2) provide valid judgement of demonstrative or performance assessment. It seems rubrics offer a way to provide the desired validity in assessing complex knowledges and capabilities, without sacrificing the need for reliability [59, 60] and (3) promote learning [57, 58]. This potential effect is focused in research on formative, self‐ and peer assessment, but also frequently mentioned in studies on summative assessment. It is assumed that the explicitness of criteria and standards are fundamental in providing students with quality feedback, and rubrics can in this way promote student learning [60, 61].

#### 3.2.1 Content Analysis of Observation Notes for the Second PD Session

Prior to the second PD session, the researchers used the physical literacy rubric shared by the HPE teachers, as a starting framework. The researchers then embedded the taxonomy shown in Table 2 into a health literacy rubric for discussion with teachers in the second PD session. During the co‐design process in the second PD session, the researchers and teachers worked together to (1) finalise a generic health literacy rubric that utilised the school's grading system; (2) that accurately described the capabilities of students' abilities and performances at each level through clear criteria and (3) and could be adapted to meet the needs of a range of formative and summative assessment tasks across Years 7–10 HPE programmes (e.g., rows could be removed if the learning activity or assessment task did not ask students to reflect).

The final consideration prompted by the teachers during the second PD session was to ensure that creativity and innovation was captured in the health literacy rubric. The impetus to foster creativity and innovation and develop critical thinking skills is at the forefront of many key policy documents underpinning Australian education. For example, the Alice Springs (Mparntwe) Education Declaration (2019) describes the overarching educational goals for young Australians. The second goal is ‘all young Australians become confident and creative individuals, successful lifelong learners, and active and informed members of the community’ (https://www.education.gov.au/alice‐springs‐mparntwe‐education‐declaration/resources/alice‐springs‐mparntwe‐education‐declaration, p. 6). This goal is reflected in the Australian curriculum with critical and creative thinking being included as a general capability (proficiency to be developed across lessons). Hence, the teachers saw the need to represent these capabilities in the health literacy rubric to align with the interactive and critical health literacy levels, with this guided by Harris, Coleman and Cook's [62] approach. The co‐designed health literacy rubric is shown in Table 4.

**TABLE 4:** Curricular model for assessing health literacy: Co‐designed health literacy rubric for use across Years 7–10 in Australian schools.

Criteria	A	B	C	D	E
Contextual understanding	Defines all key terms and makes explicit links between meanings to demonstrate holistic (sophisticated) understanding; students use creative, critical and logical reasoning in synthesing their understandings	Defines all key terms and makes some explicit links between meanings to demonstrate substantial understanding; students use some creative, critical and logical reasoning in synthesing their understandings	Defines all key terms and makes links between meanings to demonstrate sound understanding; students attempt to use some creative, critical and logical reasoning in synthesing their understandings	Define some key terms and makes limited links between meanings to demonstrate sound understanding; students are not able to use creative and critical reasoning in synthesing their understandings	Identify some key terms. Makes no links between meanings to demonstrate elementary understanding and does not attempt creative or critical synthesis
Research	Selectively accesses and navigates a broad range relevant, reliable and valid health sources; student inquiry strongly engages the commonalities, emergent themes and new questions in the research field	Selectively accesses and navigates a range relevant, reliable and valid health sources; student inquiry engages some of the commonalities, emergent themes and new questions in the research field	Accesses and navigates a range of mostly relevant, reliable and valid health sources; demonstrates some engagement with the commonalities, emergent themes and new questions in the research field	Accesses and navigates some relevant, reliable and/or valid health sources; demonstrates limited engagement with the commonalities, emergent themes and new questions in the research field	Accesses some relevant, reliable, and/or valid health sources and provide elementary engagement with the commonalities, emergent themes or new questions in the research field
Application	In creative and innovative ways applies a range of health information to novel and varied situations or settings to promote the health of self and others	Applies a range of health information to varied situations or settings to promote the health of self and others	Applies a range of health information to known and familiar situations or settings to promote the health of self	Identifies health information making limited links to known and familiar situations or settings to promote the health of self	Identifies health information. Makes elementary links to known and familiar situations or settings to promote the health of self
Critical analysis	Synthesises and evaluates health information drawing consistent justifiable conclusions, many of which are implicit	Critical analyses health information drawing mostly justifiable conclusions, some of which are implicit	Analyse health information drawing overt yet justifiable conclusions	Identifies health information demonstrating limited knowledge of reliability and/or validity and/or relevance	Identifies health information demonstrating elementary knowledge of reliability and/or validity and/or relevance
Evaluate	Uses relevant available information, determine the reliability and efficacy of a range of health information to determine reliability, validity and relevance	Analyses a range of health information to determine reliability, validity and relevance	Discusses a range of health information to describe elements of reliability and/or validity and/or relevance	Describes limited health information that has some relevance or reliability or validity only	Identifies elementary health information that has limited relevance or reliability or validity
Propose	Proposes creative, authentic and detailed action(s) to promote the health of self and others	Proposes creative and authentic action(s) to promote the health of self and others	Proposes realistic action(s) to promote the health of self	Describes limited solutions to health scenarios/situations	States elementary solutions to health scenarios/situations
Implementation	Communicates and/or executes actions to positively promote health of self and others	Communicates and/or implements actions to promote health of self and others	Communicates and/or implements actions to promote health of self	Communicates and/identity actions that demonstrate limited knowledge of promoting health of self	Communicate and/identity actions that demonstrate elementary knowledge of promoting health of self
Reflection	Critically considers thinking processes and actions (actual or proposed) to assess the impact on health of self and others	Considers thinking processes and actions (proposed) to discuss the impact on health of self and others	Reflects on actions (proposed) to discuss the impact on health of self	Describes actions drawing links to health of self	Provides elementary actions drawing links to health of self

## 4 Discussion

The aims of this study were two‐fold: (1) to develop a longer‐term PD programme for secondary school teachers to enhance their ability to plan for critical health literacy learning and (2) to co‐design with teachers a curricular model for assessing health literacy. In relation to the first aim, most learning activities in this school's HPE programmes were categorised as interactive. This is similar to previous Australian school‐based health literacy research [24, 28], which also showed that the majority of learning activities were categorised as interactive (*n* = 64% vs. 59% [28]), with a much smaller number of learning activities categorised as functional and critical (*n* = 31% vs. 17% and *n* = 4% vs. 23%, respectively [28]). Further, when the seven programmes assessed as part of the personalised PD programme, more than 70% of the activities did not promote critical health literacy. This finding is important to highlight as the AC:HPE focuses on building students' critical thinking capabilities (i.e., engage in critical inquiry to determine appropriate health information to apply to own circumstances), and emphasises the need for students to engage in learning experiences that allow them to determine the social determinants of health, and take individual and collective action to overcome, modify or work with these determinants to better their own and others' health [34, 63]. Without the opportunities to practice critical health literacy in the classroom as part of learning and assessment practices in HPE, and in this case as they work towards and achieve AC:HPE curriculum outcomes espoused in the sociocritical framing and outcomes [64], it is likely that students may not be able to develop critical health capabilities that enhance their understanding of the numerous factors determining people's health, be critical consumers of health‐related information, and make sense of health texts to take action in the context of their own and others' health needs.

It is important to note that this research study does not stipulate a need for all health education programmes to focus on critical health literacy, despite the Years 7–10 AC:HPE being designed to do so across all Year groups and health contexts [18]. Recent research has provided some evidence of longer‐term learning outcomes associated with critical health literacy without explicit teaching of critical health literacy [65]. However, without consistent practice in safe environments, such as schools and classrooms, across several health contexts (e.g., sexualities education, food and nutrition, etc.), critical health literacy knowledge and capabilities are unlikely to be fully developed. This is supported by recent research that shows: (1) that while adolescents may have high levels of digital literacy, they tend to have comparatively lower levels of critical health literacy, limiting their ability to appraise health information to take action individually and collectively [66] and (2) that although adolescents (13–15 years old) can understand complex relationships between social structures and health outcomes after engagement in comprehensive health education programmes [67], this may not equate to action or change in health behaviours. Therefore, future school‐based health literacy research needs to investigate the connection between pedagogies for enhancing critical health literacy and students' critical health literacy understandings and capabilities in the classroom and school environment through a curricular model for assessing health literacy, both in the short and longer term.

The programme analysis finding highlights that teachers in Australia have the pedagogical content knowledge to develop their students' ability to communicate, apply, understand and remember information related to health (i.e., enhance students' functional and interactive health literacy); however, they require support creating activities that promote higher critical thinking skills and designing learning experiences that allow students to take action in the classroom and beyond. This finding is unsurprising and aligns with previous research suggesting that many teachers in Australia lack the confidence and competence to teach health education to develop critical health literacy [24, 28, 68], however, teachers in this study, like the other research studies previously mentioned, are interested in participating in PD programmes that focus on developing students' health literacy.

There is a lack of PD programmes for teachers related to health education [68] and health literacy [27] and, therefore, this needs to be a consideration for HPE PD providers in Australia (e.g., ACHPER [69], national and state health organisations) and internationally. The first two face‐to‐face PD sessions focused on teachers and researchers modifying several of the interactive learning activities so that these would become critical learning activities. This often involved adding an extra question, scenario or step to the current activity to ensure that students could critically think or take action (i.e., provide a justification, design an online resource, create a rank order for effective policies). The two face‐to‐face PD sessions were also used to modify some of the functional learning activities so that they could develop students' interactive learning activities, where appropriate. Hence, teacher participants stipulated a need to implement face‐to‐face sessions initially (at least two) where personalised feedback was delivered and discussed, with future activities co‐designed with researchers. As planning and programming for this case study school was an ongoing and iterative process it was not possible to re‐analyse the HPE programmes in 2023. Nevertheless, our study supports previous findings suggesting that PD can act as a tool to support teachers to plan for health literacy learning in health education programmes [68]. Future, PD programmes need to be long‐term and sustainable (with a mix of face‐to‐face and online delivery), personalised to health education programmes designed and delivered by teachers, embed opportunities to co‐design new learning activities with teachers (particularly, focusing on enhancing students' critical health literacy), and should incorporate a second or post PD programmes analysis of health education programmes to ensure that the PD programme was effective.

In line with the second aim, the valid measurement and assessment of child and adolescent health literacy has largely been ignored in previous school‐based health literacy research focusing on Australian teachers and pedagogy [24, 26, 27, 28, 68]. In accordance with this, many researchers have recently called for advancing health literacy measurement and assessment among children and adolescents, particularly, with an emphasis on focusing on the health education curriculum in which health literacy learning in schools occurs [9, 38]. In the co‐designed curricular model of assessment that we have presented in Table 2, the teachers emphasised the need for a rubric with performance level descriptions for organising and communicating criteria, that integrated demonstrative and performance expectations for use in scoring and/or providing feedback on students' health literacy work, both in formative and summative forms. The teachers also included creative thinking into the rubric performance descriptors, based on study and policy guidelines [61]. Together, the teachers and researchers envisioned that the rubric would need to be feasible (i.e., easy to understand and use), be relevant to students and the context (e.g., school's marking criteria), would be authentic and adaptable to cover a wide range of health literacy contexts, levels, knowledge and capabilities, and has an element of open‐endedness (that can extend beyond the classroom to encompass learning across the whole school and connected with communities) [55]. Although not trialled in a HPE programme for this research study, teachers were aware that the implementation of this curricular model of health literacy would have feasibility and practical challenges. For instance, in this research study, as well as others [24, 28], findings show that teachers have found it difficult to understand the health literacy underpinning of the syllabus and provide opportunities for children to develop critical health literacy. Without ensuring that the formative learning and summative assessment tasks allow for students to demonstrate knowledge and capabilities across all three levels of health literacy (functional, interactive and critical), this rubric may be difficult for teachers (and students) to use, understand, adapt and implement. Therefore, the measurement process is only as robust as the education and health professionals who are responsible for: (1) mapping and aligning the assessment rubric to the HPE curriculum, learning outcomes and assessment tasks; (2) providing scope in assessment tasks (and rubric) for students to demonstrate/perform and be measured at all three levels of health literacy; (3) discussing the rubric and unpacking the criteria with students leading to its use with formative and summative tasks; (4) assessment of student learning through student work samples and completed learning and assessment tasks and (5) providing feedback to students that will build on their current health literacy knowledge and capabilities. This has implications for initial teacher education programmes and preservice teacher education, as well as future health promotion and health literacy programmes aiming to use primary and specialist secondary teachers to implement such programmes in schools that aim to enhance and assess students' health literacy. There needs to be substantial effort directed towards building preservice teachers, primary and secondary teachers health and assessment literacy [70, 71].

Future research is needed to validate this curricular model of assessment (i.e., rubric) using assessment tasks and student assessment response samples to consider the validity, feasibility and acceptability of the rubric for teachers and health professionals assessing health literacy and for measuring students' health literacy knowledge and capabilities. Further, this study was a small case study, used convenience sampling, and did not include audio recordings of PD sessions to capture teachers' voice in terms of the co‐design process. Hence, the evidence needs to be viewed with this in mind, as it is likely that the HPE teachers were invested in improving their teaching and assessment of health literacy and the co‐design process, and devised strategies that were contextually relevant to their setting.

## Ethics Statement

Ethical approval was gained from the University research ethics committee (HREC: 2022/732). Research was conducted in accordance with the National Statement on Ethical Conduct in Human Research (National Health and Medical Research Council, 2018). All participants provided written informed consent.

## Conflicts of Interest

The authors declare no conflicts of interest.

## Data Availability

The data that support the findings of this study are available on request from the corresponding author. The data are not publicly available due to privacy or ethical restrictions.
